# Value of parental concern and clinician’s gut feeling in recognition of serious bacterial infections: a prospective observational study

**DOI:** 10.1186/s12887-019-1591-7

**Published:** 2019-07-03

**Authors:** Urzula Nora Urbane, Dita Gaidule-Logina, Dace Gardovska, Jana Pavare

**Affiliations:** 1 Department of Pediatrics, Riga Stradins University, Vienibas Gatve 45, Riga, LV-1004 Latvia; 2 Children’s Clinical University Hospital, Vienibas Gatve 45, Riga, LV-1004 Latvia

**Keywords:** Serious bacterial infections, Children, Fever, Paediatric emergency department

## Abstract

**Background:**

Serious bacterial infections (SBI) are a significant cause of mortality worldwide. Parental concern and clinician’s gut feeling that there is something wrong has been associated with increased likelihood of developing SBI in primary care studies. The aim of this study is to assess the diagnostic value of parental concern and gut feeling at the emergency department of a tertiary hospital.

**Methods:**

This prospective observational study included children with fever attending the emergency department of Children’s Clinical University hospital in Riga between October 2017 and July 2018. Data were collected via parental and clinician questionnaires. “Gut feeling” was defined as intuitive feeling that the child may have a serious illness, and “Sense of reassurance” as a feeling that the child has a self-limiting illness. “Parental concern” was defined as impression that this illness is different from previous illnesses. SBI included bacterial meningitis, sepsis, bacteraemia, pneumonia, urinary tract infection, appendicitis, bacterial gastroenteritis, and osteomyelitis. Pearson’s Chi-Squared test or Fisher’s exact test were used to compare the variables between children with and without SBI. Positive likelihood ratio was calculated for “gut feeling”, “sense of reassurance”, and parental concern.

**Results:**

The study included 162 patients aged 2 months to 17.8 years. Forty-six patients were diagnosed with SBI. “Sense of reassurance” expressed by all clinicians was associated with lower likelihood of SBI (positive likelihood ratio 8.8, 95% confidence interval 2.2–34.8). “Gut feeling” was not significantly predictive of the patient being diagnosed with SBI (positive likelihood ratio 3.1, 95% confidence interval 1.9–5.1), The prognostic rule-in value of parental concern was insignificant (positive likelihood ratio 1.4, 95% confidence interval 1.1–1.7).

**Conclusion:**

Sense of reassurance was useful in ruling out SBI. Parental concern was not significantly predictive of SBI.

**Electronic supplementary material:**

The online version of this article (10.1186/s12887-019-1591-7) contains supplementary material, which is available to authorized users.

## Background

Fever in children is one of the most common reasons for seeking medical care. In developed countries, up to 40% of children younger than 6 months and 60% of children aged 6 months to 5 years have had fever at least once in their lifetime [1]. In most cases the underlying cause of fever is self-limiting viral infection, however, in around 4 to 25% of cases it is caused by SBIs, such as urinary tract infections (UTIs), pneumonia, osteomyelitis, cellulitis, bacteraemia, etc. [2–4]. Recognizing serious bacterial infections (SBIs) in children with fever in busy health care centres can be challenging, as fever accounts for up to 30% of patient visits in both primary care and emergency departments [5–9]. The importance of early identification of SBIs cannot be underestimated, as it is still a significant cause of mortality, even in developed countries [10]. Early diagnosis and initiation of antibacterial treatment have been emphasised in the guidelines for the management of sepsis and septic shock by Surviving Sepsis Campaign [11], as delayed administration of antibiotics and intravenous fluids in paediatric sepsis is associated with higher mortality rates [12, 13]. It is equally important identify the patients in which serious infection can be safely excluded, in order to avoid unnecessary laboratory tests and investigations, thus saving the costs as well as enabling physicians to concentrate on patients who are seriously ill.

Several clinical prediction models have been proposed for the recognition of patients with possible serious infection [14–16]. In addition to clinical signs, parental concern, defined as a statement by the parents that the particular episode of illness is different from previous episodes, has been associated with SBIs in children with fever presenting to primary care [3, 17]. In a qualitative study, the behavioural changes in children with SBIs reported by parents were drowsiness, irritability, and changed crying characteristics [18]. However, parent-reported symptoms have been found to have little predictive value for serious infection [19]. As some studies show, parental concern is affected not only to the behavioural changes and overall condition of the child, but also by their beliefs on the possible harmful effects of fever, and the support and information provided by healthcare professionals [20–22]. Also, there is scarce evidence on the performance of parental concern in tertiary health care settings.

Similarly, the “gut feeling” of the clinician (a subjective feeling that “something is wrong” even if the clinician is unsure of the basis) has shown high diagnostic value for serious illness in studies in primary care [17, 23]. Despite the fact that clinicians are encouraged to base their decisions on objective findings rather than subjective feelings, the specificity of the intuitive “gut feeling” in one of these studies was higher than that of clinical impression, the latter defined as a subjective observation that the illness was serious based on the history, observation, and clinical examination [23]. Another type of clinician instinct, sense of reassurance (feeling sure about the further management and course of the patient’s problem, even if the clinician is unsure about the precise diagnosis), has been described [24]. However, as the majority of studies on clinician instinct have been conducted in primary healthcare settings, its significance in tertiary healthcare facilities and emergency departments remains unclear.

The objectives of this study were to investigate the associations between parental concern and clinician “gut feeling” and serious bacterial infections in children with fever presenting to emergency department in a tertiary paediatric emergency department. The possible triggers of parental concern, such as behavioural changes in their child and fever phobia, were also investigated.

### Description of the clinical settings for the study

This study was conducted at the emergency department (ED) of the Children’s Clinical University Hospital in Riga, Latvia. It is attended by patients aged 0 to 18 years, with problems ranging from trauma and surgical emergencies to various childhood illnesses. The annual attendance of the ED is around 65 thousand patients, among them around 9 thousand febrile cases. Children with fever constitute for up to 22% of ED visits (excluding trauma patients).

Around 70% of all febrile patients seen in the ED undergo clinical and laboratory investigations, and the admission rate of febrile children is up to 27%. Thirty per-cent of febrile patients are discharged from the emergency department without undergoing any investigations. Most of these patients have been evaluated as clinically stable and triaged into the lowest level of urgency, where they are seen by a direct-access paediatrician in a separate section of the ED.

## Methods

### Study population

Patients aged 0 to 18 years who presented to the ED between October 2017 and July 2018 with fever (body temperature above 38 °C) or history of fever during the day of admission to the ED were enrolled in a prospective observational study. Axillary alcohol thermometers were used to assess the body temperature of patients on admission to the emergency department and during their stay in the hospital. The days of recruitment of patients were distributed evenly throughout the study period, the recruitment was conducted for approximately 4 h in each day. All eligible patients present in the emergency department (except those seen by the direct-access paediatrician) during this period were approached for the study, with the condition that the investigation results of these patients were not yet available for their attending clinician. Patients with primary and secondary immunodeficiency, or chronic illnesses that might increase the risk for infections, as well as patients transferred from other hospitals with a clear diagnosis were not enrolled.

### Gut feeling

The clinicians who first examined the patients were asked to complete a questionnaire reporting their clinical impression on the degree of severity of the child’ s illness. The “Gut feeling”, defined as an intuitive feeling that the child may have a serious illness, as well as the “Sense of reassurance”, defined as an intuitive feeling that the child has a self-limiting illness, were noted as present, or unsure/absent. The clinicians were also asked about the possible triggers of this impression, if they were able to formulate them. In addition, the physicians stated if any of the SBIs were possibly present in their patient, and to mark the presence of any of the “red flag” symptoms derived from a previously conducted systematic review [3]. The possible “red flag” signs used in this part of study were ill appearance, drowsiness, grunting, continuous crying, cyanosis, tachypnoea, shortness of breath, poor peripheral circulation, non-blanching rash, seizures, hypotension, loss of consciousness. This survey was conducted immediately after the first physical examination of the patient, before the data on laboratory investigations or imaging diagnostics were available to the clinician.

### Parental concern

The data from the parents of patients enrolled in this study were collected via questionnaire including questions on the behavioural changes observed during the febrile episode, their concern about the child’s condition, and additional questions on their beliefs on the management and effects of fever. Information on the age and education of the parents, number of children in the family, and the child’s previous illnesses was also collected. Parental concern, defined as the impression that this illness is different / more severe than the previous illnesses, was marked as either present, or unsure/absent. The parents were approached preferably after physical examination but within 48 h of admission, whenever the child’s condition allowed them to concentrate on the questionnaire.

### Outcome definitions

The primary outcome of interest was the presence of any of SBIs, defined as bacterial meningitis, sepsis, bacteraemia, pneumonia (positive consolidation on chest X-ray), urinary tract infection (positive urine culture and microscopy), bacterial gastroenteritis (positive bacterial pathogen in stool), appendicitis, and osteomyelitis [25]. The secondary outcomes were rates of admission, antibacterial treatment, and readmittance within 72 h.

The patients were followed up until formulation of diagnosis, to exclude or confirm development of SBI. Most of the patients (and all the patients with SBIs) underwent laboratory and/or imaging diagnostics, however for ethical reasons the study did not require additional testing that was not requested by the clinicians involved in treating the patient.

### Data analysis

The studied population was described via descriptive statistics. The presence of parent-reported behavioural changes, parental concern, gut feeling, and sense of reassurance was compared between the patients with and without SBI using the chi-squared test (critical value 3.8) or Fisher’s exact test, as appropriate. A *p* value of less that 0.05 was considered significant. Associations between parent-reported behavioural changes and development of SBI were evaluated by Odds Ratio. Positive and negative likelihood rates for “gut feeling”, “sense of reassurance”, and parental concern in relation to development of SBI were calculated.

## Results

### Participants

Between October 2017 and July 2018, 6451 febrile children were evaluated in the ED. Out of these patients, 1869 were triaged to be evaluated by the direct-access paediatrician, and the remaining 4582 children were seen at the main part of the ED and were considered eligible to participate in the study. The recruitment occurred over 88 days during the study period. During the selected study dates, 2007 children with fever presented to the ED, 1478 of whom were seen at the main part of the ED and were eligible for the study. All patients who were present during the recruitment hours and satisfied the conditions described in Methods were approached, and each day one to six patients were enrolled.

Overall, 266 patients were approached. Twenty-four patients were excluded as the clinicians did not manage to fill the clinician questionnaire before viewing the investigation results. Forty-six parents refused participation in the questionnaire, and 33 failed to submit the completed questionnaire within the specified time period. One patient was excluded as the evidence on the parental questionnaire suggested it was filled by the child and not the parent. In total, the study included 162/4582 patients (3.5% of eligible presentations) (162/1478 or 11% of eligible presentations on the recruitment dates). Of them 86 (53.1%) were boys. The age range of the patients was 2 months to 17.8 years, with the median age 43.5 months. Twenty-two patients were younger than 1 year, and 80 patients were 1 to 5 years old.

Of the clinicians who participated in the study, 85 (52.5%) were certified paediatricians with 5 to 53 years of experience, the rest of them were paediatric residents with one to 4 years of experience. Most of the parents (88.3%) who took part in the survey were mothers (*n* = 143), 15 were fathers, and in four cases the survey was filled in by another guardian accompanying the child during admission. The age of the mothers participating in the study ranged from 22 to 56 years (median 34 years), the age of participating fathers was 25 to 52 years (median 33 years). The demographic data on the study population and participants, as well as qualification of the clinicians can be viewed in detail in Additional file 1.

### Outcomes

Serious bacterial infection was present in 46 patients. The final diagnoses of the patients are presented in Table 1.

**Table 1:** Final diagnoses of the patients enrolled in the study

	Absolute number	Percentage
Serious bacterial infection present	46	28.4%
Urinary tract infection	7	4.3%
Sepsis / bacteraemia	3	1.9%
Pneumonia	28	17.3%
Acute osteomyelitis with bacteraemia	1	0.6%
Bacterial meningitis with bacteraemia	1	0.6%
Bacterial gastroenteritis	3	1.9%
Acute appendicitis	2	1.2%
Bacterial soft tissue infection (phlegmon)	1	0.6%
Serious bacterial infection absent	116	71.6%
Upper respiratory tract infections (adenovirus, bocavirus, RSV or unspecified)	28	17.3%
Influenza	13	8.0%
Croup	2	1.2%
Acute otitis media	3	1.9%
Scarlet fever	3	1.9%
Pharyngitis, tonsillitis	27	16.7%
Viral gastroenteritis	8	4.9%
Viral lower respiratory tract infections	9	5.6%
Aseptic meningitis	2	1.2%
Viral syndrome	14	8.6%
No definite diagnosis	7	4.3%

Almost all patients with SBI were hospitalized, except five patients with pneumonia who were discharged from the ED after less than 24 h, with initiated antibacterial treatment. The parents of one other patient with urinary tract infection refused hospitalization. None of these patients were readmitted to the ED within the same episode of illness. The median duration of hospitalization for the patients with SBI was 4 days, with the maximum of 31 days. Five patients were hospitalized in the intensive care unit for 1 to 2 days before transfer to the wards.

Among patients who did not develop SBI, 47 patients were prescribed antibacterial treatment, and 57 patients were hospitalized, with the median duration of stay being 3 days. Three of the patients in this group were readmitted to the hospital after discharge, the reason for readmission in both cases was development of symptoms of acute gastroenteritis. The data on clinical outcomes can be viewed in detail in Additional file 1.

### Diagnostic value of clinician instinct

The presence of clinician’s “gut feeling” was significantly more common in children who developed SBI than in those who did not, as was “sense of reassurance” in the cases with no SBI. However, the prognostic value of “gut feeling” in ruling in or ruling out the possibility of being diagnosed with SBI in the study population was not significant. The likelihood of the patient being diagnosed with SBI was higher when “gut feeling” was expressed by certified paediatricians than when stated by paediatric residents. Sense of reassurance was associated with decreased likelihood of having SBI. The rule-out value of sense of reassurance was not significant. The sensitivity, specificity, predictive values, positive and negative likelihood ratio, Pearson’s chi-square and *p* values of “gut feeling” and parental concern are displayed in Table 2. The diagnostic value of sense of reassurance for the absence of SBI is examined in Table 3.

**Table 2:** Diagnostic value of “gut feeling” and parental concern for the presence of SBI

	SBI present	SBI absent	Sensitivity, %	Specificity, %	Predictive value, %		Positive and negative likelihood ratio (95% CI)		Chi-Square (*P* value)
					positive	negative	LR+	LR-	
“Gut feeling” of all clinicians									
Present	23	19	50.0	83.6	54.8	80.8	3.1 (1.9–5.1)	0.6 (0.4–0.8)	19.4 (0.000)
Absent	23	97							
“Gut feeling” of certified paediatricians									
Present	9	5	39.1	91.9	64.3	80.3	4.9 (1.8–13.0)	0.7 (0.5–0.9)	(0.002)^a^
Absent	14	57							
“Gut feeling” of paediatric residents									
Present	14	14	60.9	74.0	50.0	81.6	2.4 (1.3–4.1)	0.5 (0.3–0.9)	8.5 (0.004)
Absent	9	40							
Parental concern (“different illness”)									
Present	38	67	82.6	41.2	36.1	85.4	1.4 (1.1–1.7)	0.4 (0.2–0.8)	8.2 (0.004)
Absent	8	47							

^a^Fisher’s exact test was applied when the number of subjects in one of the cells in the 2 × 2 contingency table was less than 5

**Table 3:** Diagnostic value of sense of reassurance for the absence of SBI

	SBI absent	SBI present	Sensitivity, %	Specificity, %	Predictive value, %		Positive and negative likelihood ratio (95% CI)		Chi-Square (*P* value)
					positive	negative	LR+	LR-	
Sense of reassurance^a^ in all clinicians									
Present	44	2	38.3	95.7	95.7	38.2	8.8 (2.2–34.8)	0.7 (0.6–0.8)	18.5 (0.000)
Absent	71	44							
Sense of reassurance^a^ in certified paediatricians									
Present	25	1	40.3	95.7	96.2	37.3	9.3 (1.3–64.6)	0.6 (0.5–0.8)	10.2 (0.001)
Absent	37	22							
Sense of reassurance^a^ in paediatric residents									
Present	19	1	35.9	95.7	95.0	39.9	8.25 (1.2–58.0)	0.7 (0.5–0.8)	8.2 (0.04)
Absent	34	22							

^a^Sense of reassurance (expectation of absence of SBI) in case of absent SBI was considered as true positive

The presence of the previously listed “red flag” signs was associated with gut feeling (OR (95% CI) = 9.4 (3.4–25.5), *p* = 0.000). These “red flag” signs were noted in 21 out of the 23 cases (91.3%) in which the clinician expressed gut feeling, and the child was diagnosed with SBI. Similarly, parental concern was associated with gut feeling (OR (95% CI) = 3.4 (1.4–8.4), *p* = 0.01).

### Diagnostic value of parental concern and observations

Parental concern (“different illness”) was significantly more commonly expressed by parents of children who developed SBI (as reflected in Table 2), however its value in predicting SBI in children with fever was poor. None of the parent-reported symptoms and behavioural changes listed in the survey (rapid or superficial breathing, grunting, moaning, rejection of favourite toys or activities, inconsolable crying, screaming, irritability, drowsiness, refusal of food or drinks, decreased urination) had a significant association with developing SBI. None of these behavioural changes were also identified as a significant trigger to parental concern. There were no differences in the parental ability to predict SBI depending on number of children in the family, parental age or level of education. The information on parental age range, education level, and observations during the episode of illness can be viewed in detail in the Additional file 1.

Parental beliefs on effects and proper management of fever showed elements of fever phobia in the study population. Ninety-seven parents (59.9%) stated a belief that fever itself is indicative of serious illness, 45 parents believed that other symptoms must be considered as well when evaluating the severity of illness, only 14 parents did not believe that fever is indicative of serious illness. However, no association was found between the belief that fever is indicative of serious illness and parental concern. Fourteen parents gave antipyretics to their children in case of elevated body temperature that did not reach 38 °C, most of the parents (80.9%, *n* = 131) gave medication when the temperature was between 38 and 38.5 °C, only 14 parents allowed the body temperature of their child to increase above 38.5 °C, regardless of how the child was feeling. 52 parents (32.1%) believed that body temperature above 39 °C is life-threatening to their child; 28 parents (17.2%) attributed these dangerous effects to temperature between 39.5 °C and 39.9 °C, and 60 parents (37.0%) – to temperature above 40 °C.

## Discussion

### Summary of main findings

In this prospective observational study sense of reassurance, an impression that the child has a self-limiting illness, was predictive of the absence of SBI. “Gut feeling” that the child has a serious illness, though more commonly expressed by clinicians evaluating the patients who were later diagnosed with SBI, was of limited prognostic value, especially when expressed by paediatric residents rather than their senior colleagues.

Parental concern (“different illness”) was more commonly expressed by parents of patients with SBI, however its value in prognosing SBI in children with fever was of little significance. Parent-reported symptoms and behavioural chances were also poor prognostic factors for SBI. There were elements of fever phobia among the participating parents.

### Comparison with existing literature

Several qualitative studies have shown that non-analytical, intuitive reasoning plays a significant role in decision making process of a doctor [26–29]. These studies explain that intuitive feelings, which emerge quickly and with little effort, aid the diagnostic process in sometimes complex and unclear clinical situations when relying on objective findings and facts alone would make it difficult to decide on the most appropriate immediate actions [30]. Yet the number of studies focusing on the accuracy of clinician instinct is very small, which complicates the introduction of the concept of gut feeling in applied medicine and medical education.

Most studies on the performance of clinician instinct focus on “gut feeling” of something wrong (sense of alarm) [17, 23], the diagnostic accuracy of sense of reassurance is yet to be examined. Qualitative research studies have found that sense of reassurance often lets doctors avoid unnecessary investigations and rather refer the patients for careful observation, although objective findings and rational arguments for taking action are relied upon more than their intuition [27, 28]. In our study we found that sense of reassurance was useful in recognizing cases when serious bacterial infections were unlikely. More studies in assessing its diagnostic value would provide a better insight in its applicability in medical situations when the diagnosis is unclear and objective signs suggesting serious illness are absent.

Currently all studies assessing the diagnostic accuracy of “gut feeling” (sense of alarm) have been conducted in primary care. The positive likelihood ratio and specificity of gut feeling derived from this study population was significantly lower than obtained in one Belgian study published in 2012 [23]. In our study, the positive likelihood ratio of gut feeling expressed by certified clinicians was nearly 5, but overall analysis did not yield a significant result. This can be partially explained by the lack of continuity of care, which is an important factor leading to gut feeling in case of serious illness [29]. As this study was conducted in an emergency department of a tertiary hospital, the participating clinicians had in most cases never seen the particular patient before, so distinguishing abnormal behaviour or appearance from the one natural for the patient in a state of well-being or non-serious illness was not possible. In our study there were significant differences between the positive likelihood ratios of “gut feeling” when expressed by senior and junior clinicians, in contrast to the previously mentioned Belgian study [23], where its performance was equal. In the same Belgian study, another term, “clinical impression”, a subjective observation that the illness is serious based on objective information (history, observation, examination), was distinguished from the more intuitive “gut feeling”. The specificity of gut feeling was higher than clinical impression, suggesting that more holistic approach to evaluation of a patient’s leads to a better recognition of serious illness. The discrimination of the two terms was not applied to our research, as clinical impression was replaced with the presence or absence of “red flag” signs. It was evident that clinical presentation was suggestive of serious illness in great majority of cases when gut feeling correctly identified SBI, which together with the role of experience leads to a conclusion that “gut feeling”, although defined as an intuitive feeling, may be reliant on conscious evaluation rather than unconscious instincts. The subtle integration of cognitive processes into instinctual evaluation has been considered in other studies [27, 29]. For example, in a qualitative, focus group study among general practitioners, most of the participants believed that gut feeling can be taught, as after years of experience the analytical process had become automatic, providing an intuitive feeling that an illness might be serious, or in other cases non-serious, without a lengthy process of reasoning [27]. The ability to form intuitive discriminations is stated as characteristic to “expertise”, the final stage of “The Five-Stage Model of Adult Skill Acquisition” [31].

Contrary to the evidence in primary care [3, 17], parental concern was not strongly associated with increased likelihood of the child being diagnosed with SBI, though it was more commonly expressed by the parents of the latter. This can be due to the definition of parental concern as the illness being different from the child’s previous illnesses. This definition was derived from a qualitative study in primary care [18] and may not be applicable to emergency departments and tertiary hospitals, to which the child is referred to in cases of more severe illnesses than have been managed in primary care. Elements of fever phobia were also evident in the study population, but their contribution to developing parental concern as defined in this study was not clear. Similarly to the findings of another study conducted in a paediatric assessment unit at University Hospitals Coventry and Warwickshire in the UK, parent-reported symptoms and behavioural changes had no predictive value in recognizing SBI [19].

### Limitations of study

There are several limitations of this study, which we are aware of. The recruitment process, though distributed evenly during the study period, was not inclusive of all febrile patients presenting to ED, and the sample size of patients who provided informed consent was small. The prevalence of serious bacterial infections in the study sample was nearly 30%, which does not represent the overall prevalence of SBI in the study site. However, as the patients were recruited prospectively, the final diagnosis at the time of recruitment was unknown. Patients receiving intravenous fluids and awaiting blood test results were more likely to stay longer at the emergency department and thus their parents were more prone to voluntarily participate in the parental survey and complete it by the end of their stay. As a result, some parents failed to submit the survey and were therefore excluded, and the patients classified as lower risk and discharged after examination or rapid antigen testing were scarcely enrolled (the study population included four such patients).

This study was focused on assessing the diagnostic value of clinician instinct and parental concern in recognizing serious bacterial illness, however, viral infections with moderate to severe course are also common in ED and were present among the study population, such as in case of viral meningitis and bronchiolitis with respiratory insufficiency, or viral gastroenteritis with dehydration. As parental concern and gut feeling of a possible serious illness are not discriminative between viral and bacterial infections, the false positive responses cannot always be associated with poor ability to identify SBI.

This was the first study conducted for assessment of performance of clinician instinct at the study site. The survey given to clinicians was discussed amongst the paediatricians working at the emergency department prior its application in the study. A decision to omit the assessment of clinical impression was made as the definitions of both gut feeling and clinical impression were found to be confusing and to increase the time necessary to fill the survey. Therefore, the presence of “red flag” signs was used instead to evaluate the impact of clinical presentation on the formation of gut feeling. In the Belgian observational study [23] the two definitions were discerned without difficulty, which may indicate that the concept of gut feeling may be understood differently between doctors from different cultural backgrounds.

### Clinical implications of the study results

Our study suggests that sense of reassurance decreases the likelihood of the patient being diagnosed with SBI. However, it should be approached with caution if objective findings or other data suggest that SBI is possible, to avoid any missed cases. “Gut feeling” of a possible serious illness, when felt by a clinician after examining a child with fever at the emergency department, did not prove to an effective tool for predicting serious bacterial infection when analysed alone. Nevertheless, it was more commonly expressed by doctors in cases where SBI were later identified. Therefore, if the doctor feels a sense of alarm, the patient still needs to be approached with care, and necessary investigations for exclusion of SBI should be performed.

Parental concern, although not associated with noticeably higher likelihood of SBI, should also be considered as alarming, but must be evaluated together with other factors increasing the likelihood of SBI, such as the “red flag” signs, and clinician’s gut feeling.

The small study sample reduces the applicability of the results to the whole population attending paediatric emergency department with complaints of fever, consecutive enrolment with a larger study sample would be preferable and provide more reliable results.

## Conclusion

In the study population, clinician instinct as sense of reassurance was useful in ruling out serious bacterial infection. “Gut feeling” (sense of alarm) alone was not significantly predictive of SBI. Parental concern, if defined as the feeling that the illness of the child is different from previous febrile illnesses, did not significantly increase the likelihood of SBI. A study with larger sample size must be conducted to increase the reliability and applicability of the results to the general population of febrile children attending emergency department.

## Additional file

Additional file 1: Study population, participants and results of parental and clinician questionnaires. Information on the demographic data and outcomes of the study population, as well as data collected in the parental and clinician questionnaires that were analysed for this article. (XLSX 36 kb)

## Data Availability

All data analysed during this study are included in this published article or can be viewed on a separate dataset spreadsheet (Additional File 1).

## Abbreviations

CI: Confidence interval
ED: Emergency department
LR-: Negative likelihood ratio
LR+: Positive likelihood ratio.
OR: Odds ratio
RSV: Respiratory syncytial virus
SBI: Serious bacterial infections
